# Cancer risk awareness and screening uptake in individuals at higher risk for colon cancer: a cross-sectional study

**DOI:** 10.1136/bmjopen-2016-013833

**Published:** 2016-12-20

**Authors:** Hamideh Salimzadeh, Faraz Bishehsari, Alireza Delavari, Gilda Barzin, Mohammad Amani, Azam Majidi, Alireza Sadjadi, Reza Malekzadeh

**Affiliations:** 1 Digestive Oncology Research Center, Digestive Disease Research Institute, Tehran University of Medical Sciences, Shariati Hospital, Tehran, Iran; 2 Division of Gastroenterology, Department of Internal Medicine, Rush University Medical Center, Chicago, Illinois, USA

**Keywords:** Family cancer, compliance, colonoscopy, awareness, colorectal cancer screening

## Abstract

**Objective:**

We aimed to measure cancer knowledge and feasibility of a screening colonoscopy among a cohort of individuals at higher risk of colon cancer.

**Methods:**

This study was conducted as part of an ongoing screening cohort, in which first degree relatives (FDRs) of patients with colon cancer are invited to participate in a free of charge screening colonoscopy. We enrolled 1017 FDRs in the study between 2013 and 2014 measuring their data on demographics, cancer knowledge and colonoscopy uptake. A p value of <0.05 was considered statistically significant.

**Results:**

The relative's mean age was 48.7 years. Only about 28% of FDRs were aware of their increased risk for cancer, near 35.0% had ever heard about colonoscopy with 22% aware of the correct age to start screening. Comparing cancer knowledge of FDRs at high risk versus those at moderate risk, we recorded non-significant differences (p>0.05). Almost two-thirds of FDRs expressed willingness to undergo a colonoscopy and 49.2% completed the procedure, of which 12.8% had advanced neoplasm.

**Conclusions:**

Our data indicated that remarkable numbers of FDRs were not still informed of their cancer risk or never received a physician recommendation for screening. The desirable uptake at first invitation, which would be higher over successive invitations, supports the feasibility of a family-based recruitment approach for early screening. This has promising implications to introduce targeted screening colonoscopy into the healthcare system in Iran and other developing nations.

**Strengths and limitations of this study**

- Our data are applicable to other developing countries with no mass screening programmes.
- We approached the index patients to reach their relatives, as were not allowed to directly contact their relatives due to medical ethics.
- As expected at each stage of the recruitment process some factors impaired the enrolment which might potentially lead to selection bias. Nevertheless, we asked participant first degree relatives (FDRs) to inform all their eligible relatives to take part in the study and enrolled a mean of three FDRs (range=1–7) per index patient who could be representative of their possibly at-risk yet non-participant relatives.
- In our study colonoscopy as standard of care was offered free of charge. Then, it remains unclear whether our data would be applicable if individuals are asked to contribute to the cost of colonoscopy in a real-world setting.

## Introduction

Colorectal cancer (CRC) is a major cause of cancer death worldwide.^1^ Colonoscopy is known as an effective screening method in the prevention of CRC through detecting and removing precancerous polyps.^2^ Current guidelines recommend first degree relatives (FDRs), parents, siblings or child, of patients with colon cancer to start screening colonoscopy at younger ages.^3^ Relative risk of developing cancer among family members of patients with CRC is twofold to threefold greater than the general population,^4^ thus, likely gaining the greatest benefit from early CRC screening.^5^

Studies addressing risk awareness in relatives of patients with CRC have shown that these individuals usually perceive a greater overall risk of developing CRC in a relative sense,^6–8^ in contrast to other studies which suggest poor knowledge about CRC risk factors^9^ and screening tests in these high-risk people.^10^ ^11^ Although people with an elevated risk of CRC were found to be more compliant to screening guidelines compared with average-risk individuals,^12^ they are also underusing CRC screening.^9^ Data of more than 6800 participants in the USA showed that only 45% of individuals with a strong family history of CRC had undergone CRC screening and over 50% were not aware that they should be screened at an early age compared with the moderate-risk people.^11^

According to the Asia Pacific consensus, the implementation of CRC screening is recommended in regions with a high incidence of the disease (>30 per 100 000).^13^ Although the overall incidence of CRC is rising in Iran, it is not among high incidence countries; therefore, mass screening for CRC is not justified yet.^14^ However, our prior data have shown a risk of family history for patients with early-onset CRC in Iran.^15^ A desirable alternate approach, therefore, would be targeting people with an increased risk of CRC for a nationwide screening plan.^9^ To the best of our knowledge, there are no data on cancer risk knowledge and screening in individuals at familial risk for CRC in Iran and it is unknown whether they are aware of the implications of having a positive family history of CRC for early screening. However, cancer risk awareness and colonoscopy uptake need to be assessed before introducing any targeted screening programmes into the health system in Iran. This is the first family-based study in Iran that aimed to measure cancer risk knowledge and the feasibility of a screening colonoscopy among a cohort of FDRs. Our data will provide important data for future health services and behavioural interventions with regard to improving early screening in these high-risk individuals.

## Methods

### Study design and setting

This study was conducted as part of an ongoing population-level screening cohort, in which relatives of patients with CRC are invited to participate in a screening programme in the Digestive Disease Research Institute (DDRI), affiliated to Tehran University of Medical Sciences (TUMS). All eligible participants were offered a free of charge colonoscopy conducted under conscious sedation. The study protocol was approved by the Institutional Review Board of the DDRI, TUMS. We measured cancer risk knowledge and colonoscopy uptake in FDRs and intended to determine whether their cancer knowledge varies by the level of familial risk.

### Enrolment process

We adopted a family-based multistep recruitment approach. The initial sample comprised patients with histologically verified malignancy in their colon or rectum, hereafter referred to as ‘index patients’, reported between 2010 and 2013 by the national cancer registry system. This registry as a collaborative effort of the Deputy of Health in the TUMS and the International Agency for Research on Cancer have been established in Iran since 1967,^16^ providing detailed data about patients with cancer.

We contacted index patients after obtaining permission from the TUMS Ethics Committee, as it is not ethical to directly approach the relatives of CRC cases for patient autonomy and privacy. In case index patients died, their closest living relatives (eg, spouse) were interviewed. During the initial contact, the objectives of the study were explained to the index patients or living relatives in detail. Then, those who were willing to participate in the study were asked to share the contact information of the DDRI screening centre with their possibly at-risk relatives and encourage them to call or attend the screening centre. Male and female FDRs were enrolled if they were 40 years old or 10 years prior to the earliest diagnosis in their family, whichever comes first; those with a personal history of CRC or inflammatory bowel disease (ie, Crohn's disease or ulcerative colitis) were excluded from the study. Participant FDRs were provided with enough information about the significance of CRC and undergoing a colonoscopy. FDRs who agreed to have or scheduled a colonoscopy received detailed in-person instruction plus an educational pamphlet about bowel preparation. Informed consent was obtained from all individual participants included in the study.

### Measures

Data collection started through phone calls and was completed via in-person interviews once FDRs attended the screening centre. The basic characteristics of index cases, for example, age at which the index patient was first diagnosed with cancer and contact details were extracted from the cancer registry data set. The FDRs' data included age, gender, educational level, and marital status, job, medical insurance, CRC symptoms (eg, change in bowel habits, rectal bleeding), type of relation with the index patient (siblings, parents and off-spring), and number of relatives affected with CRC in a family. We also assessed test-specific awareness of faecal occult blood test (FOBT), colonoscopy/sigmoidoscopy, knowledge of CRC, knowledge source (physician or the media), willingness to undergo and the uptake of colonoscopy among participants.

Interviewers provided participants with a brief explanation about each screening test and then asked if they had ever heard about the corresponding test. In order to measure FDR's knowledge of CRC (ie, risk factors, symptoms and screening tests), we used a previous questionnaire which contained 11 true–false statements (Cronbach’s α=0.81) as follows:^17^ ‘the risk of developing CRC increases with age’, ‘people with a family history of CRC are at higher risk’, ‘some polyps can turn into cancer over time’, ‘early CRC often has no symptoms’, ‘CRC may cause rectal bleeding or blood in the stool which may make it look dark’, ‘CRC may cause cramping or abdominal pain’, ‘CRC may cause a change in bowel habits, for example, diarrhoea and/or constipation that lasts for more than a few days’, ‘CRC may cause unexplained weight loss’, ‘all people ≥50 years should get screened regularly for CRC’, ‘finding and removing polyps early helps prevent cancer’, ‘people with a family history of CRC may get tested at age 40 or earlier’.

In order to determine whether the FDR's cancer knowledge is affected by the level of familial risk, we grouped them into high-risk versus moderate-risk groups. For this analysis, the high-risk group included individuals who had more than one patient with CRC in family or had one index patient <60 years of age at diagnosis, or presented at least one symptom suggestive of CRC (eg, rectal bleeding, abdominal pain, etc). FDRs not fulfilling the high-risk criteria were defined as moderate risk. Advanced neoplasm was defined as presence of cancer or high-risk adenomas (adenomas sized ≥10 mm and/or with a villous component, and/or with high-grade dysplasia) in colonoscopy findings.

### Statistical analysis

Continuous variables were reported as the mean and SD and the qualitative variables as numbers and percentages. The χ^2^ or Fisher's exact tests were applied to describe differences between the subgroups. All statistical tests were two tailed and a p value of <0.05 was considered statistically significant. Stata/MP software V.12 was used for analyses.

## Results

### Sample characteristics

**Figure 1:**
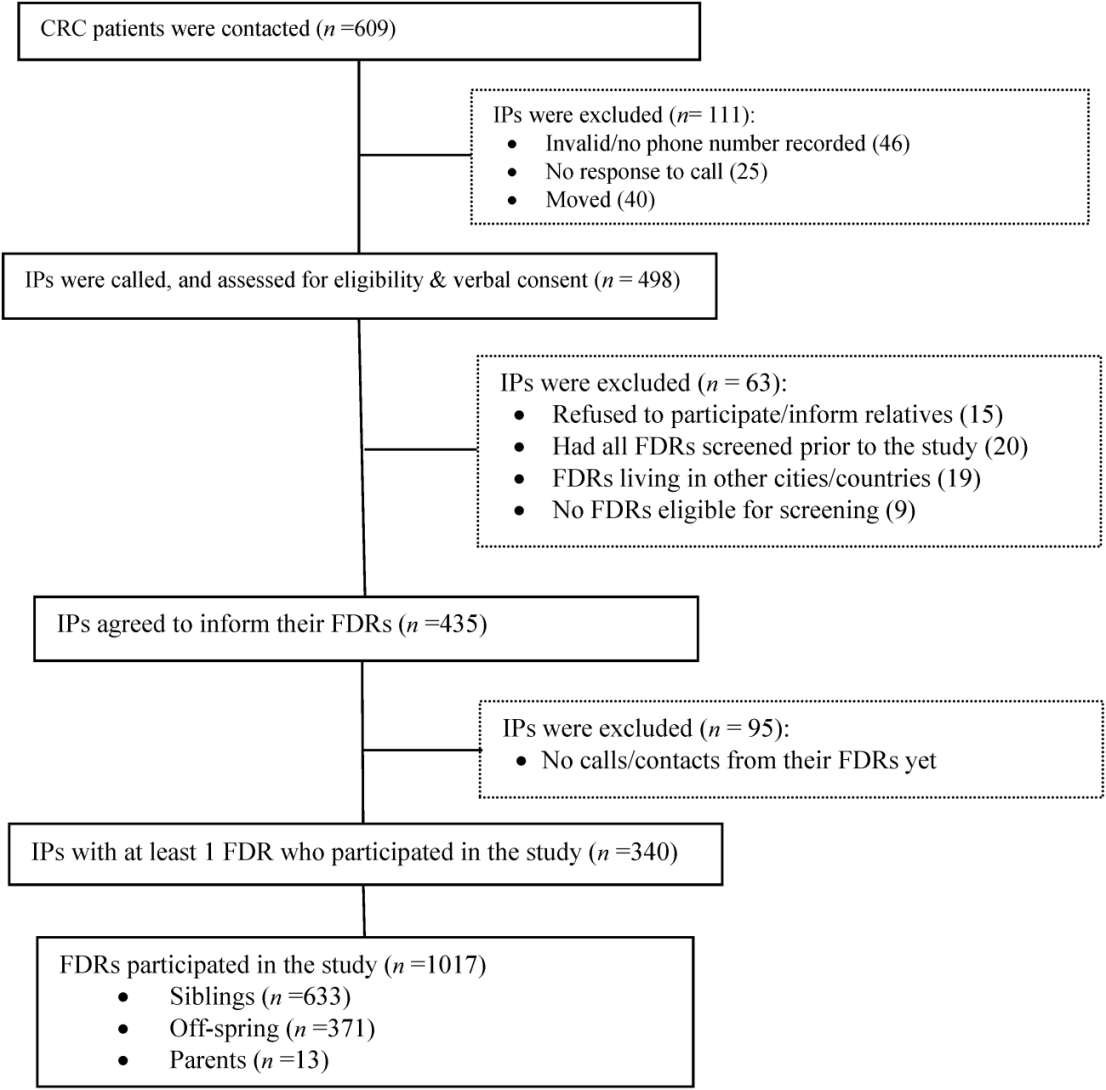
Flow chart of the enrolment. CRC, colorectal cancer; FDR, first-degree relative; IP, index patient.

A total number of 609 patients with CRC were identified by the cancer registry of the TUMS from 2010 to 2012. Of these, 46 (7.6%) were not reachable due to invalid phone numbers and 65 (10.7%) had moved or changed their phone number or were not reachable on phone after three attempts to calls at different times. Overall, 81.8% of the index patients (498/609) responded to our call; of these, 15 index patients (3.0%) did not wish to disclose their disease information with their siblings; 4.0% (20/498) stated that all their eligible relatives had already undergone a colonoscopy; 3.8% (19/498) noted that their FDRs lived in other cities or countries; and there were 9 index patients (1.8%) who had no child/siblings and/or their parents were too old for screening (ie, >86 years). Overall, 435 of 498 (87.3%) index patients agreed to inform their relatives. Finally, a total of 1017 FDRs belonging to 340 index patients (mean 3.0 relatives per patient) personally called and/or attended our screening centre during 2013–2014 (figure 1).

Characteristics of index patients and their FDRs are shown in table 1. The mean age (SD) of the index patients at diagnosis of cancer was 55.0±11.5 years. In 61.0% (n=620) of FDRs, the index patient was younger than 60 years at the time of diagnosis. About 13% of FDRs reported having at least two members affected with CRC in their family. In relation to the type of familial relationship of the FDRs with the index patient, 62.2% (n=633) were siblings and 37.8% (n=384) were parents/off-spring of the index patient (table 1).

**Table 1:** Characteristics of the participants (n=1017)

Index patient age at diagnosis (mean±SD)	55.0 (11.5)
Index patient <60 years at diagnosis, n (%)	620 (61.0)
Number of affected members in family, n (%)	
One	888 (87.3)
Two or more	129 (12.7)
FDR's relationship with index patient, n (%)	
Siblings	633 (62.2)
Parents/off-spring	384 (37.8)
FDR's age (mean±SD)	52.0±10.6
Gender—female, n (%)	594 (58.4)
Marital status, n (%)	
Married	925 (91.0)
Single/widowed/divorced	92 (9.0)
Employment, n (%)	
Employed/self-employed	438 (43.1)
Retired	171 (16.8)
Housekeeper/jobless	408 (40.1)
Years of schooling, n (%)	
≥12	633 (62.2)
<12	384 (37.8)
Had medical insurance, n (%)	1017 (100.0)
Symptoms, n (%)	
None	828 (81.4)
Change in bowel habits	98 (9.6)
Cramping/abdominal pain	30 (3.0)
Rectal bleeding/melena	59 (5.8)
Unexplained weight loss	2 (0.2)

FDR, first-degree relative.

The mean age (SD) of the FDRs was 52.00±10.6 years (range=22–86) and only <1% (n=10) of them were above the recommended age for screening (>75 years.). Of the total sample, over 58% were women, 91% were married and all were insured. About 43.1% (n=438) were employed or self-employed and 62.2% had 12 or more years of education. Overall, about 20% of the FDRs reported having already one symptom suggestive of CRC, for example, rectal bleeding, abdominal pain, etc (table 1).

### Cancer knowledge and risk level

Overall, only 4.1% (n=42) of the FDRs provided correct responses to all the 11 items regarding cancer knowledge. Detailed data on the FDR's cancer risk knowledge are shown in table 2. About 28% (n=284) answered that they were aware of the increased risk for the development of CRC in relatives. Advanced age was cited as a risk factor of CRC by 26.2% of the FDRs and 25.4% knew that polyps may turn into cancer over time. While a minority of the participants (22.5%) noted that early CRC often has no symptoms, only 10.4% were able to correctly recognise the alarming symptoms of CRC (table 2).

**Table 2:** Specific knowledge on colon cancer and screening tests (n=1017)

	Total*(*n*=1017)	Moderate risk*(*n*=283)	High risk*(*n*=734)	p Value†
People with a family history of CRC are at higher risk	284 (27.9)	73 (25.8)	211 (28.8)	0.34
The risk of developing CRC increases with age	267 (26.2)	70 (24.7)	197 (26.8)	0.49
Some polyps can turn into cancer over time	258 (25.4)	69 (24.4)	189 (25.8)	0.65
Early CRC often has no symptoms	229 (22.5)	60 (21.2)	169 (23.0)	0.53
CRC may cause some symptoms‡	106 (10.4)	22 (7.8)	84 (11.4)	0.09
Heard of FOBT	168 (16.5)	45 (15.9)	123(16.7)	0.74
Heard of sigmoidoscopy/colonoscopy	356 (35.0)	100 (35.3)	256 (34.9)	0.89
People with a family history of CRC may get tested at age 40/early	220 (21.6)	54 (19.1)	166 (22.6)	0.22
All people ≥50 years should get screened regularly for CRC	191 (18.8)	48 (17.0)	143 (19.5)	0.35
Finding and removing polyps early helps prevent cancer	202 (19.9)	53 (18.7)	149 (20.3)	0.57

*Number (percentage) of yes/correct responses.

†Moderate risk versus high risk.

‡Rectal bleeding/blood in the stool, cramping/abdominal pain, a change in bowel habits, unexplained weight loss.

CRC, colorectal cancer; FOBT, faecal occult blood test.

We found that only 16.5% and 35.0% of the FDRs had ever heard about FOBT and sigmoidoscopy/colonoscopy, respectively. About 22% of FDRs knew that they should receive screening at age 40 or earlier and nearly 19% stated that all adults aged 50 years and older should get screened regularly for CRC. Almost 20% of FDRs were aware that early polyp removal helps prevent CRC. Most FDRs (72.2%) were found to be at higher risk for CRC, that is, already presenting at least one CRC-related symptom or having a young index patient or several members affected in their family. We recorded no significant differences for cancer knowledge between high-risk FDRs and those at moderate risk for CRC (p>0.05; table 2).

### Awareness source and colonoscopy uptake and results

The main source of awareness was physicians as reported by 42.0% of the FDRs with only 1.1% receiving information from TV or the internet. About 65% (n=657) of FDRs expressed their willingness to have a screening colonoscopy within the next 6 months, of which 76.1% (500/657), corresponding to 49.2% (500/1017) of the total sample, completed the procedure (table 3). The mean age of screened relatives was 47.5±10.8 (range 22–75) years, and women comprised 54.0% (n=270) of them. Advanced neoplasia was identified in 13.4% (67/500) of the screened FDRs and cancer in 1.8% (n=9), 7 at stage II and 2 at stage III (data not shown).

**Table 3:** Source of awareness, and use of colonoscopy (n=1017)

	Number (%)
Source of awareness	
None	579 (56.9)
Physician	427 (42.0)
TV/the internet	11 (1.1)
Willingness to have a colonoscopy	
No	360 (35.4)
Yes	657 (64.6)
Colonoscopy use	
No	517 (50.8)
Yes	500 (49.2)

## Discussion

The present study indicates, for the first time in Iran, that FDRs of patients with CRC enrolled in a population-based screening programme are clearly lacking in basic knowledge about CRC and screening tests. We found that nearly three-fourth of individuals with an established elevated risk for CRC were not aware of their CRC risk and the significance of undergoing screening tests, comparable with those outlined in American, Canadian and also Asian studies.^7^ ^9^ ^11^

Studies from populations with high rates of CRC have demonstrated that FDRs of patients with CRC perceive themselves to be a greater risk of CRC^6–8^ and that their awareness of the actual risks posed by the disease contributes to a favourable screening uptake.^18–20^ It was noteworthy that less than one-third of our FDRs were aware of their increased risk for the development of CRC, which was below the estimates (44–51%) from studies in Canada^9^ ^10^ where similar to Iran there is no mass screening for CRC. Only a minority of FDRs (26%) were aware that advanced age is a risk factor of CRC and a quarter knew that polyps may turn into cancer over time, compatible with results of the studies mentioned.^9^ Although a reasonable proportion of the participants (∼20%) were found to be symptomatic, the vast majority of the respondents did not know that the alarming symptoms could associate with CRC. These results are in contrast with the recent study suggesting greater knowledge of CRC symptoms and risk factors among Chinese FDRs.^7^

Public awareness about CRC screening is on the rise in developed countries including those with no mass screening for CRC^9^ whereas, the majority of FDRs in our study reported that they had not heard about sigmoidoscopy/colonoscopy and near 80% were not aware that early polyp removal helps cancer prevention. This indicates that our FDRs do not clearly understand the key benefits of CRC screening and early polyp removal. Additionally, only one-fifth of our participants knew about the correct age to start screening in high-risk people, compared with the estimates (<50%) of other series.^10^ ^11^ The risk of developing CRC increases further for relatives of early-onset CRC cases or members with two or more CRCs in family.^21^ These FDRs, however, found to have no greater cancer knowledge versus those at moderate risk of CRC, which contradicts the findings of other studies.^22^ ^23^

In Iran, patients with CRC during the disease diagnosis or treatment process are routinely encouraged by their physicians to pass on the information of CRC risk and early screening to their at-risk relatives. Though the FDRs of patients with CRC would have had the opportunity to receive cancer information from their physicians and other possible sources, less than half of FDRs (42%) noted their physicians as the main source of awareness and screening recommendation. Physicians are considered as the primary eligible candidates to recommend CRC screening to at-risk individuals;^9^ this essential role, however, might be overlooked to some extent as reflected in the previously published literature.^17^ ^24^ Thus, the gaps in FDRs' knowledge might be attributed, in part, to the poor knowledge transfer between the patient and physician in their communication process. This highlights the importance of providing relatives with sound recommendations about CRC screening by physicians or health providers in primary care settings. Adopting such policies could prevent the rising CRC burden^14^ in developing nations with no current mass screening programmes for the disease.

Cancer is a complex topic and people need guidance in realising the significance of cancer prevention and overcoming cancer myths and misconceptions. Mass media is known to be an effective tool of publicising cancer awareness,^25^ ^26^ whereas only 1% of our FDRs had obtained awareness from the media. This implies the poor performance of our mass media with regard to risk communication and cancer awareness.

There are no data about screening behaviours of the FDRs in Iran, but a recent study suggested an overall poor uptake (11%) of CRC screening tests in our average-risk people.^27^ In our study, the expressed willingness to use colonoscopy (∼65%) was impressive, which is the first step of the procedure leading up to actual uptake. The uptake of colonoscopy (∼50%) in our FDRs is greater than those shown by studies from countries with high rates of CRC,^8^ ^22^ ^28^ yet lower than the estimates (>60%) of a few reports.^12^ ^29^ On the contrary, half of the FDRs failed to have a colonoscopy possibly due to low-risk perception, negative attitudes towards colonoscopy or other reasons which may have led to limited access to the procedure.^17^ ^24^ ^27^ ^30^ This highlights that these individuals may need more resources or motivation to proceed with colonoscopy testing. Further studies are, therefore, warranted to explore the important barriers or predictors of colonoscopy use as screening test among Iranian high-risk population. Studies on FOBT suggested that repeated invitations to screening or reminders can significantly engage a remarkable number of previous non-responders.^31^ This suggests that repeated invitation rounds and effective communication could translate into an increase in the uptake of screening colonoscopy as well, particularly among families with an increased risk for this malignancy who reject first screening invitation.

Owing to medical ethics, we were not allowed to directly approach the relatives of the index patients, so we adopted a feasible approach by asking the index patients to inform their relatives to contact or attend our screening centre. As expected at each stage of the recruitment process some factors impaired the enrolment which might potentially lead to selection bias (figure 1). Nevertheless, we enrolled a mean of three FDRs (range=1–7) per index patient who could be representative of their possibly at-risk yet non-participant relatives and asked participant FDRs to inform all their eligible relatives to take part in the study. In our study, colonoscopy as standard of care was offered free of charge. Then, it remains unclear whether our data would be applicable if individuals are asked to contribute to the cost of colonoscopy in a real-world setting. However, only a minority (4.0%) of average-risk adults has cited cost as a major barrier to CRC screening.^27^ Moreover, medical insurance in Iran, with coverage of about 85%, would cover costs of colonoscopy for symptoms, family history of CRC and surveillance.^32^

This is the first study in Iran, a traditionally low-incident area for CRC with current rising rates of the disease,^33^ ^34^ that targets individuals who are known to have an elevated risk of CRC using cancer registry data. Our data suggest that a remarkable number of the FDRs are not still informed of their cancer risk and available screening methods. This reflects that the focus areas of CRC control programmes would be conducting awareness campaigns about CRC risk and screening tests and training primary care physicians/health providers about the significance of CRC screening.

## Conclusion

Our favourable uptake of colonoscopy as the choice of screening modality over one screening invitation supports the feasibility of a family-based recruitment approach for CRC screening among Iranian high-risk people in real-world settings. This could have promising implications to offer targeted screening colonoscopy as a standard of care to family members of patients with CRC as part of our healthcare services and could be applicable to other developing countries with no current mass screening programmes that are experiencing similar epidemiological transitions of CRC.
